# VEGF Expression in Head and Neck Squamous Cell Carcinoma: Association with TNM Staging and Tumor Progression

**DOI:** 10.3390/cimb48070725

**Published:** 2026-07-16

**Authors:** Cristina Stefania Dumitru, Alina Cristina Barb, Antonia Armega Anghelescu, Dorin Novacescu, Marina Rakitovan, Cristian Silviu Suciu, Flavia Zara

**Affiliations:** 1 Department II of Microscopic Morphology, Discipline of Histology, “Victor Babes” University of Medicine and Pharmacy Timisoara, E. Murgu Square, No. 2, 300041 Timisoara, Romania; cristina-stefania.dumitru@umft.ro (C.S.D.); novacescu.dorin@umft.ro (D.N.); marina.rakitovan@umft.ro (M.R.); cristian_suciu@umft.ro (C.S.S.); flavia.zara@umft.ro (F.Z.); 2 Angiogenesis Research Center, “Victor Babes” University of Medicine and Pharmacy, 300041 Timisoara, Romania

**Keywords:** VEGF, head and neck squamous cell carcinoma, angiogenesis, immunohistochemistry, TNM staging, clinicopathological features, tumor progression

## Abstract

Conventional TNM staging in head and neck squamous cell carcinoma (HNSCC) does not fully capture the molecular heterogeneity driving tumor aggressiveness. Identifying accessible biomarkers that complement staging is therefore a clinical priority. This retrospective study evaluated immunohistochemical VEGF expression in 50 histopathologically confirmed HNSCC cases and examined its association with TNM staging and clinicopathological parameters. VEGF expression was assessed using a semi-quantitative immunoreactivity score (IRS; range 0–12; median 5; IQR 3–8). High VEGF expression (IRS ≥ 4) was identified in 64% of cases and was significantly associated with advanced tumor stage (III–IV; *p* = 0.032), higher T stage (T3–T4; *p* = 0.041), and regional lymph node metastasis (*p* = 0.018). Multivariate logistic regression confirmed nodal status as an independent predictor of high VEGF expression (OR = 2.85; 95% CI 1.12–7.24; *p* = 0.028). These findings indicate that VEGF expression is associated with adverse pathological features and markers of tumor progression in HNSCC. In the absence of survival data, prognostic conclusions cannot be drawn; prospective studies with survival endpoints are required to establish the clinical utility of VEGF as a prognostic biomarker.

## 1. Introduction

Head and neck squamous cell carcinoma (HNSCC) accounts for approximately 90% of all head and neck malignancies and represents the seventh most common cancer worldwide, with an estimated 890,000 new cases and 450,000 deaths annually [1,2]. Despite diagnostic and therapeutic advances—including surgery, radiotherapy, chemoradiotherapy, and immune checkpoint inhibition—5-year overall survival remains highly variable (25–90%) depending on tumor stage, anatomical subsite, HPV status, and biological behavior [3]. A central clinical challenge is that TNM staging, while essential for treatment planning, does not adequately reflect the molecular heterogeneity that drives differences in tumor aggressiveness, metastatic potential, and treatment response [4,5]. This gap underscores the need for accessible tissue biomarkers that can complement conventional staging. Tumor angiogenesis plays an important role in the growth, invasion, and metastatic dissemination of solid tumors, including HNSCC, with over 90% of HNSCCs expressing angiogenic factors [6,7]. Among the various pro-angiogenic factors, vascular endothelial growth factor (VEGF), particularly VEGF-A, has been identified as the single most important regulator of tumor neovascularization [8,9]. VEGF promotes endothelial cell proliferation, migration, and survival, primarily through VEGF receptor 2 (VEGFR-2), leading to the formation of an abnormal vascular network that supports tumor expansion and facilitates metastatic spread [8,9]. Increased VEGF expression has been associated with enhanced tumor aggressiveness, higher metastatic potential, and poorer prognosis in multiple malignancies [8,10].

In HNSCC, the tumor microenvironment is characterized by hypoxia and inflammatory signaling, both of which contribute to the upregulation of VEGF through hypoxia-inducible factor-1α (HIF-1α)–mediated pathways [11,12,13]. This molecular interplay enhances angiogenic activity and may influence tumor progression and resistance to therapy. Consequently, VEGF has emerged not only as a potential biomarker of tumor aggressiveness but also as a therapeutic target in oncology, with VEGF pathway inhibitors being investigated in combination with conventional therapies [14,15].

Immunohistochemical (IHC) evaluation of VEGF expression in tumor tissues provides a practical and widely accessible method for assessing angiogenic activity in routine clinical practice [16,17]. Two major meta-analyses have investigated the prognostic significance of VEGF in HNSCC. Kyzas et al. (2005), in a meta-analysis of 12 studies (*n* = 1002 patients), demonstrated that VEGF-positive patients had a 1.88-fold higher risk of death at 2 years (95% CI, 1.43–2.45; *p* < 0.001), although VEGF overexpression was not significantly associated with lymph node metastasis [16]. Zang et al. (2013), in a larger meta-analysis of 47 studies, confirmed that VEGF overexpression was significantly associated with worse overall survival (HR, 1.89; 95% CI, 1.61–2.22) and progression-free survival (HR, 1.68; 95% CI, 1.33–2.12) [18]. However, the reported results remain heterogeneous, particularly regarding the association of VEGF with TNM staging and its prognostic significance across different subsites and stages [16,17,18]. Some studies have found that VEGF and microvessel density correlate with clinical stage, while others have reported that VEGF expression is down-regulated during head and neck tumorigenesis, or that angiogenic biomarkers are prognostic only in early-stage disease [19,20,21]. This variability highlights the need for further studies in well-defined patient cohorts to clarify the clinical relevance of VEGF expression.

Against this background, the present study provides an IHC-based evaluation of VEGF expression in a well-characterized Romanian cohort, applying a standardized scoring approach, and investigates its association with TNM staging and clinicopathological features to determine which pathological parameters are independently associated with elevated VEGF expression [16,17,18,19,20,21]. Unlike most prior series, which report VEGF-clinicopathological associations without a reproducible scoring framework or multivariable adjustment, this study combines a semi-quantitative IRS system with confirmed interobserver reliability (κ = 0.78) and multi-variate logistic regression to identify which parameters are independently associated with VEGF expression, rather than relying on univariate correlations alone—an analytic step not consistently reported in the existing literature.

## 2. Materials and Methods

### 2.1. Study Design and Patient Selection

This retrospective cohort study included patients diagnosed with head and neck squamous cell carcinoma (HNSCC), whose tumor specimens were collected at the Otorhinolaryngology Clinic of Timișoara between October 2019 and October 2020 and subsequently processed at the Department of Histology, “Victor Babeș” University of Medicine and Pharmacy, Timișoara. Archival formalin-fixed, paraffin-embedded (FFPE) tissue samples from a previously established doctoral cohort were reviewed. Of an initial pool of 67 tumor specimens, 50 histopathologically confirmed HNSCC cases fulfilled all inclusion criteria and constituted the final study population. The 17 excluded cases were removed for the following reasons: insufficient residual tumor tissue in the paraffin block (*n* = 8), incomplete clinicopathological records including missing TNM staging data (*n* = 5), and absence of representative tumor areas within the section suitable for IHC evaluation (*n* = 4). Cases were included if they had histopathological confirmation of HNSCC, adequate FFPE tumor tissue, and complete corresponding clinicopathological data. Cases were excluded if tissue material was insufficient or degraded, if representative tumor areas were absent, or if clinical or pathological records were incomplete. The final sample size of 50 cases was determined by the availability of a pre-existing, well-annotated archival cohort meeting strict criteria for tissue adequacy and clinicopathological completeness, rather than by an a priori power calculation; the resulting statistical power for the principal association (approximately 72%; see Section 4) is acknowledged as a limitation warranting cautious interpretation of borderline findings. Although the 17 excluded cases were removed on the basis of tissue adequacy and docu-mentation completeness rather than tumor stage, a systematic difference in stage distribution between excluded and included cases cannot be fully verified retrospectively; this is addressed further as a limitation in Section 4. Several anatomical subsites (larynx, oral cavity, oropharynx, hypopharynx, and other sites) were analyzed together in order to pre-serve adequate statistical power given the overall sample size. We acknowledge that these subsites differ in underlying molecular biology, HPV association, and prognosis, and that pooling them may introduce biological heterogeneity; anatomical site was therefore not used as a stratification variable in the multivariate model, and subsite-specific analyses in larger, dedicated cohorts are needed to determine whether the associations reported here hold uniformly across subsites.

### 2.2. Clinical and Pathological Data Collection

Clinical and pathological data were obtained from the patients’ medical records and the doctoral database, ensuring consistency between histopathological findings and clinical staging parameters. The following variables were collected and analyzed: demographic data (age at diagnosis and sex); tumor-related characteristics including anatomical site (larynx, oropharynx, hypopharynx, oral cavity, sinonasal region, or nasopharynx); histopathological parameters including tumor differentiation grade (G1, G2, G3) according to standard morphological criteria; TNM classification based on the American Joint Committee on Cancer (AJCC) staging system; and clinicopathological features associated with tumor aggressiveness and progression, as available in the database.

Tumor staging was performed according to the AJCC 8th edition, incorporating primary tumor size and extension (T), regional lymph node involvement (N), and distant metastasis (M), where applicable. For statistical purposes, cases were further stratified into early-stage (I–II) and advanced-stage (III–IV) disease [22].

Data on smoking history, alcohol consumption, and HPV/p16 status were not uniformly available across all patient records and were therefore not included in statistical analyses. Survival and recurrence data were available for only a subset of patients owing to incomplete follow-up in this retrospective cohort, and were consequently excluded to avoid systematic bias. These constitute acknowledged limitations of the study.

All clinical and pathological data were reviewed and cross-validated to ensure accuracy and completeness prior to statistical analysis.

### 2.3. Tissue Processing and Histopathological Examination

The study was performed on archival formalin-fixed, paraffin-embedded (FFPE) tissue blocks obtained from the previously established cohort. All specimens had been originally collected and processed according to standard histopathological protocols, including fixation in 10% buffered formalin, dehydration, clearing, and paraffin embedding.

For the purposes of the present study, FFPE tissue blocks were retrieved from the histopathology archives and sectioned at 3–4 μm thickness using a microtome. The sections were mounted on adhesive glass slides for subsequent histopathological and immunohistochemical analyses.

Routine histopathological evaluation was based on hematoxylin–eosin (H&E)-stained sections, which were reviewed to confirm the diagnosis of head and neck squamous cell carcinoma and to assess morphological characteristics, including tumor differentiation, growth pattern, and invasion features.

Tumor grading was performed according to established morphological criteria, classifying tumors as well-differentiated (G1), moderately differentiated (G2), and poorly differentiated (G3), based on keratinization, nuclear pleomorphism, and mitotic activity.

All cases were independently reviewed by experienced pathologists, and representative tumor areas were selected for subsequent VEGF immunohistochemical analysis.

### 2.4. Immunohistochemical Analysis of VEGF

Immunohistochemical (IHC) staining for VEGF was performed using the Bond Max Autostainer (Leica Biosystems, Newcastle upon Tyne, UK) in a fully automated manner. The primary antibody was anti-VEGF-A (mouse monoclonal, clone VG1, Leica Biosystems; manufacturer-validated for human tissue; dilution optimized per laboratory protocol). Heat-induced epitope retrieval was performed using Bond Epitope Retrieval Solution 1 or 2 (Leica Biosystems) for 20 min, selected based on antibody optimization. Endogenous peroxidase was blocked with 3% hydrogen peroxide for 5 min. Primary antibody incubation was carried out at room temperature for 30 min. Visualization was achieved with the Bond Polymer Refine Detection System (Leica Biosystems) for 15 min, using 3,3-diaminobenzidine dihydrochloride (DAB) as chromogen and hematoxylin as counterstain. A section incubated without primary antibody served as the negative control. Endothelial cells within each section served as internal positive controls, given their constitutive VEGF expression. Staining was performed in two sequential automated runs, and results were consistent across runs, confirming assay reproducibility.

### 2.5. Evaluation of VEGF Expression

VEGF expression was assessed semi-quantitatively by two independent pathologists blinded to clinical and pathological data. The evaluation was performed on at least five high-power microscopic fields (×400) per case, with attention to the invasive front and regions of maximal staining intensity. Only cytoplasmic granular staining in tumor cells was considered specific and included in the scoring; nuclear staining, when present, was regarded as non-specific and excluded. VEGF immunoreactivity was quantified using the immunoreactivity score (IRS), calculated as the product of staining intensity (IS) and the proportion of positive tumor cells (PS): IRS = IS × PS (range 0–12). Staining intensity was graded as: 0, absent; 1, weak; 2, moderate; 3, strong. The proportion of positive cells was graded as: 0 = 0%; 1 = 1–10%; 2 = 11–50%; 3 = 51–80%; 4 ≥ 80%. For statistical analysis, VEGF expression was dichotomized into low expression (IRS 0–3) and high expression (IRS ≥ 4). This threshold was selected based on the distribution of IRS values observed in our cohort, where IRS = 4 represented the natural inflection point separating cases with absent-to-weak expression from those with moderate-to-strong expression, as reflected in the score distribution shown in Section 3.2. Discrepant scores between the two pathologists were resolved by consensus review. Interobserver agreement was assessed using Cohen’s kappa coefficient (κ = 0.78), indicating substantial agreement. Endothelial cells within each section served as internal positive controls given their constitutive VEGF expression, and were not included in tumor cell scoring. Staining in stromal cells, fibroblasts, and inflammatory cells was similarly excluded from the IRS evaluation.

### 2.6. Statistical Analysis

Statistical analysis was performed using SPSS software (Statistical Package for the Social Sciences, version 26.0, IBM Corp., Armonk, NY, USA). Continuous variables were expressed as mean ± standard deviation (SD) or median (interquartile range), depending on data distribution, while categorical variables were presented as frequencies and percentages.

Associations between VEGF expression and clinicopathological parameters were evaluated using the Chi-square test or Fisher’s exact test, as appropriate. Statistical significance was set at *p* < 0.05 (two-tailed). Univariate logistic regression was performed to identify candidate predictors of high VEGF expression. Variables achieving *p* < 0.10 in univariate analysis, together with those of established clinical relevance (tumor stage, nodal status, histological grade), were entered into a multivariate logistic regression model. Variable selection was constrained by the events per variable (EPV) criterion: with 32 outcome events (high VEGF expression), a maximum of three predictor variables was included to maintain EPV ≥ 10 (achieved EPV = 10.7). Multicollinearity was assessed using Variance Inflation Factors (VIF). Model fit was evaluated with the Hosmer–Lemeshow goodness-of-fit test, and explanatory power was estimated using the Nagelkerke pseudo-R^2^. Results are reported as odds ratios (OR) with 95% confidence intervals (CI).

## 3. Results

### 3.1. Patient Characteristics

A total of 50 patients with histopathologically confirmed HNSCC were included in the study. The mean age at diagnosis was 61.4 ± 9.8 years (range: 42–78 years), with a predominance of male patients (*n* = 36, 72%) compared to females (*n* = 14, 28%).

Tumor localization was heterogeneous, with the larynx being the most frequently affected site (*n* = 18, 36%), followed by the oral cavity (*n* = 14, 28%) and oropharynx (*n* = 10, 20%). Less frequent sites included the hypopharynx (*n* = 5, 10%) and other anatomical regions (*n* = 3, 6%).

Histopathological grading revealed a predominance of moderately differentiated tumors (G2) (*n* = 26, 52%), followed by poorly differentiated tumors (G3) (*n* = 15, 30%) and well-differentiated tumors (G1) (*n* = 9, 18%).

According to TNM staging (AJCC 8th edition), most patients presented with advanced disease. Early-stage tumors (stage I–II) were identified in 14 cases (28%), whereas advanced-stage tumors (stage III–IV) were observed in 36 cases (72%). Regional lymph node metastases (N+) were present in 30 patients (60%), while distant metastases at diagnosis were uncommon. These clinicopathological characteristics are summarized in Table 1.

The high proportion of advanced-stage disease (72% stage III–IV) reflects the late-stage diagnosis pattern commonly observed in the Romanian clinical setting, where delayed healthcare presentation is frequent. This cohort composition should be considered when interpreting the associations described below.

### 3.2. VEGF Expression in HNSCC

VEGF immunohistochemical expression was observed in the majority of HNSCC cases, displaying a predominantly cytoplasmic granular staining pattern in tumor cells. The evaluation was performed on representative tumor areas, with particular emphasis on the invasive front and regions of maximal staining intensity, as described in Section 2. Only cytoplasmic staining was considered specific for VEGF expression.

A heterogeneous pattern of VEGF expression was identified across the study cohort, both in terms of staining intensity and the proportion of positive tumor cells. The immunoreactivity score (IRS), calculated as the product of staining intensity and the percentage of positive cells, ranged from 0 to 12. The distribution of IRS values demonstrated a wide variability, with a median IRS of 5 (interquartile range: 3–8), reflecting intertumoral heterogeneity in VEGF expression.

Analysis of the individual IRS components revealed that moderate to strong staining intensity (IS 2–3) was observed in the majority of cases, while the proportion of positive tumor cells varied considerably, with several tumors showing diffuse positivity (>50% of cells).

For statistical purposes, VEGF expression was dichotomized into low and high expression groups. The IRS ≥ 4 threshold for high expression corresponds to the natural inflection point in the distribution of IRS observed in our cohort. Low VEGF expression (IRS 0–3) was identified in 18 cases (36%), whereas high VEGF expression (IRS ≥ 4) was observed in 32 cases (64%).

**Figure 1:**
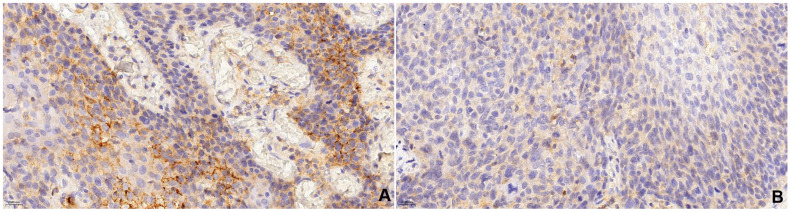
Immunohistochemical expression of VEGF in HNSCC (brown staining). (**A**) Moderate cytoplasmic staining in tumor cells, corresponding to high VEGF expression. (**B**) Weak cytoplasmic staining in tumor cells, corresponding to low VEGF expression. Original magnification ×400.

Tumors with high VEGF expression frequently exhibited more heterogeneous staining patterns, with areas of intense positivity interspersed with moderately stained regions. In addition, increased VEGF expression was often associated with more prominent cytoplasmic staining at the invasive tumor front, supporting its role in tumor angiogenesis and progression. The distribution of VEGF IRS is summarized in Table 2. Representative examples of low and high VEGF expression are illustrated in Figure 1.

### 3.3. Correlation Between VEGF Expression and Clinicopathological Features

Statistical analysis demonstrated significant associations between VEGF expression and key clinicopathological features.

High VEGF expression was significantly associated with advanced tumor stage (stage III–IV), being observed in 26 out of 36 advanced-stage cases (72.2%), compared to 6 out of 14 early-stage cases (42.9%) (χ^2^ = 4.62, *p* = 0.032).

Similarly, a significant correlation was identified between VEGF expression and lymph node status. High VEGF expression was more frequently observed in patients with nodal metastases (N+), being present in 23 out of 30 cases (76.7%), compared to 9 out of 20 cases (45.0%) without nodal involvement (χ^2^ = 5.64, *p* = 0.018).

Regarding tumor differentiation, high VEGF expression was more frequently observed in poorly differentiated tumors (G3) (80%), compared to moderately (57.7%) and well-differentiated tumors (55.6%); however, this association did not reach statistical significance (*p* = 0.089).

No statistically significant associations were found between VEGF expression and patient age or sex (*p* > 0.05). These associations are detailed in Table 3.

Further analysis demonstrated that high VEGF expression was significantly associated with parameters of tumor progression, particularly tumor size and local invasion, as reflected by T stage. Tumors classified as T3–T4 exhibited a significantly higher frequency of high VEGF expression compared to T1–T2 tumors (*p* = 0.041), as shown in Table 3.

These findings support the association between increased VEGF expression and more advanced local tumor progression.

### 3.4. Predictors of High VEGF Expression

Based on the univariate associations reported in Section 3.3, variables with potential clinical relevance, including tumor stage, nodal status, and histological grade, were subsequently included in a multivariate logistic regression model.

Multivariate logistic regression (3 variables, 32 events; EPV = 10.7, meeting the conventional ≥ 10 threshold) demonstrated that lymph node involvement (N+) remained a significant predictor of high VEGF expression (OR = 2.85; 95% CI: 1.12–7.24; *p* = 0.028), while tumor stage showed a trend toward significance (*p* = 0.061). The model demonstrated acceptable fit (Hosmer–Lemeshow *p* = 0.41) and moderate explanatory power (Nagelkerke R^2^ = 0.24). Variance Inflation Factors were 2.1 (tumor stage) and 2.3 (nodal status), confirming acceptable multicollinearity. As a sensitivity check, a model including nodal status alone yielded consistent results (OR = 2.91; 95% CI: 1.14–7.43; *p* = 0.026). Results are summarized in Table 4.

## 4. Discussion

The present study evaluated VEGF expression in HNSCC by immunohistochemistry and its relationship to TNM staging and clinicopathological parameters. The main finding—that high VEGF expression is significantly associated with advanced tumor stage, regional nodal involvement, and higher T stage—is consistent with the biological role of VEGF in promoting angiogenesis, invasion, and metastatic spread. Below, we critically discuss each key finding in relation to our own data and to the existing evidence. In our cohort, high VEGF expression was identified in 64% of cases, reflecting the high prevalence of angiogenic activity in HNSCC. This rate is consistent with, though at the higher end of, values reported in prior series (range approximately 40–75%), likely reflecting the predominance of advanced-stage tumors in our cohort [5,20,23]. While this cohort composition may have amplified the observed associations, it also accurately represents the clinical reality of HNSCC presentation in our institution, where late-stage diagnosis is common. A comprehensive review by Dumitru and Raica (2023) confirmed that VEGF-A remains the most extensively studied member of the VEGF family in HNSCC and plays a key role in tumor angiogenesis and progression [23]. VEGF is known to promote endothelial cell proliferation and vascular permeability, contributing to the formation of an aberrant tumor vasculature that facilitates tumor growth and metastatic dissemination [24]. Furthermore, Siemert et al. (2021) demonstrated that pre-therapeutic plasma VEGF levels serve as an independent prognostic biomarker in HNSCC, with patients having VEGF plasma levels 26 ng/L showing superior nodal control, loco-regional control, progression-free survival, and overall survival in two independent cohorts [25].

A key finding of our study is the significant association between VEGF expression and advanced tumor stage (III–IV), as well as T stage. From our own data, the gradient is clear: 72.2% of stage III–IV cases showed high VEGF expression versus 42.9% of stage I–II cases (*p* = 0.032). We interpret this as reflecting VEGF-driven angiogenesis being more pronounced in larger, more invasive tumors—consistent with the role of VEGF in facilitating the metabolic demands of bulky tumor masses. However, we acknowledge that the predominance of advanced-stage cases in our cohort may have amplified these associations, and replication in a more stage-balanced population is warranted. Similar findings have been reported in previous studies [5,26]. Tse et al. (2007) showed that advanced T stage and strong VEGF immunoreactivity were independent adverse predictors for overall and disease-free survival [5]. However, the literature remains heterogeneous. Schlüter et al. (2018) found that in laryngeal SCC, high VEGF expression was prognostic specifically in early-stage disease but not in late-stage tumors [19]. This stage-dependency is relevant to interpreting our findings: our cohort is weighted toward advanced disease, and VEGF associations in early-stage patients may differ. Another important result of our study is the identification of lymph node involvement (N+) as an independent predictor of high VEGF expression. In our data, 76.7% of N+ cases showed high VEGF expression versus 45.0% of N0 cases (*p* = 0.018), and this association survived multivariate adjustment (OR = 2.85, *p* = 0.028). We propose that VEGF-A contributes to lymph node metastasis primarily through VEGFR-2-mediated hemangiogenesis and increased peritumoral vascular permeability, which may facilitate tumor cell intravasation; direct lymphangiogenic signaling is more specifically attributed to VEGF-C and VEGF-D acting via VEGFR-3, which were not assessed in the present study. Any contribution of VEGF-A to lymphatic spread in our cohort is therefore likely indirect, mediated through hemangiogenic remodeling of the tumor microenvironment rather than direct lymphatic vessel induction. While the earlier meta-analysis by Kyzas et al. (2005) did not find a significant overall association between VEGF and nodal status (RR: 1.20; *p* = 0.087) [16], individual studies—including Mineta et al. (2000) and O-charoenrat et al. (2001)—did report significant associations [4,27]. The discrepancy may reflect heterogeneity in study design, VEGF measurement methods, and tumor subsite distribution. Of note, potential collinearity between tumor stage and nodal status in the multivariate model was assessed: VIF values of 2.1 and 2.3 are below the threshold of concern, and a sensitivity model with nodal status alone yielded consistent results (OR = 2.91, *p* = 0.026), supporting the robustness of this finding. Srivastava et al. (2014) also reported elevated serum VEGF-A in N+ patients (*p* = 0.004) [28], and the meta-analysis by Zang et al. (2013) confirmed VEGF overexpression is associated with worse overall survival and progression-free survival [18]. The lack of a statistically significant association between VEGF expression and tumor grade in our study is in line with several previous reports [5,20]. We interpret this finding as reflecting the conceptual distinction between histological differentiation—a morphological characteristic determined by keratin production and nuclear atypia—and angiogenic activity, which is driven by hypoxia and metabolic stress rather than differentiation status. A moderately differentiated tumor with a hypoxic core may exhibit higher VEGF expression than a poorly differentiated but well-vascularized one. This biological dissociation may explain why grade-VEGF correlations are inconsistent across the literature [29,30]. This biological dissociation may instead reflect distinct, only partially overlapping molecular pathways underlying differentiation and angiogenesis; because no survival data were collected, this observation should be regarded as hypothesis-generating rather than evidence of independent prognostic value. To contextualize our findings within the broader literature, Table 5 provides a comparative summary of key studies on VEGF expression in HNSCC.

From a biological perspective, the upregulation of VEGF in HNSCC can be explained by tumor hypoxia and activation of hypoxia-inducible factors, particularly HIF-1α. Hypoxic conditions within the tumor microenvironment stimulate VEGF expression, leading to increased angiogenesis and adaptation to adverse conditions [6,30]. Hu et al. (2023) detailed the molecular mechanisms of HIF-1α activation in HNSCC, highlighting the roles of reactive oxygen species and the PI3K/Akt/mTOR cascade in HIF-1α stabilization and consequent VEGF upregulation [11]. Mukhopadhyay et al. (2024) demonstrated that high HIF-1α/VEGF protein expression correlated with poor clinical outcomes in HNSCC [31]. This hypoxia-driven mechanism not only sustains tumor growth but also contributes to treatment resistance, as hypoxic tumors show reduced responsiveness to radiotherapy and chemotherapy. Bao and Liao (2025) showed that HIF-1α promotes radioresistance via Notch1 pathway activation [32], Hill et al. (2024) identified HIF-pathway-mediated autophagy as a key driver of radioresistance under mild hypoxia [33], and Wiechec et al. (2022) linked hypoxia to epithelial–mesenchymal transition and cancer stem cell phenotype in HNSCC [34]. Taken together, these data suggest that the high VEGF expression observed in our advanced-stage cases may in part reflect a hypoxic tumor microenvironment, with implications not only for angiogenesis but also for treatment response.

The association between high VEGF expression and markers of aggressive disease observed in our cohort has direct clinical relevance in the context of available VEGF-targeted therapies. While the present study does not include survival data and prognostic conclusions cannot be drawn, the pathological correlates of high VEGF expression identified here—advanced stage and nodal spread—are precisely the disease characteristics that define patients most likely to be considered for anti-angiogenic treatment. The E1305 trial (Argiris et al., 2019) demonstrated that adding bevacizumab to platinum-based chemotherapy improved progression-free survival (6.0 vs. 4.3 months; *p* = 0.0014) and overall response rate in recurrent/metastatic HNSCC, although overall survival was not significantly prolonged and toxicity was increased [35]. A phase 1/2 trial by Adkins et al. (2024) combining ramucirumab with pembrolizumab achieved an objective response rate of 55% [36], leveraging the rationale that VEGF suppresses anti-tumor immunity by inhibiting T-cell trafficking and expanding immunosuppressive cell populations [36,37]. Hyytiinen et al. (2021) concluded in a systematic review of 38 trials that angiogenesis inhibitors showed limited benefit as monotherapy but encouraging results in combination [38]. The ongoing EA3202 trial evaluates atezolizumab plus bevacizumab in the post-immunotherapy setting [39], and microenvironment data further support correlation between VEGF activity and immune infiltration in HNSCC [22,40,41]. Collectively, these data reinforce the rationale for VEGF pathway characterization in HNSCC and support the translational relevance of the associations identified in our study. Several limitations of this study should be carefully considered. First, the sample size of 50 patients from a single center limits the statistical power and generalizability of the findings. Post hoc power analysis indicates approximately 72% power to detect the observed effect size (OR ≈ 2.85) at α = 0.05, falling marginally below the conventional 0.80 threshold. Second, the retrospective design introduces potential selection bias; the 17 excluded cases (8 due to insufficient tissue, 5 due to incomplete records, 4 due to non-representative sections) could theoretically introduce selection effects. Although exclusion was based on tissue and documentation adequacy rather than tumor stage per se, a systematic difference in stage distribution between excluded and included cases cannot be fully verified retrospectively, and this remains an inherent limitation of the archival cohort design. Third, HPV/p16 status was not assessed in this cohort, which is a significant limitation particularly for the 10 oropharyngeal cases (20%), in whom HPV status is a major independent determinant of prognosis and potentially of VEGF expression. Fourth, smoking and alcohol history data were incompletely recorded and could not be included in the analysis. Fifth, survival data were available for only a subset of patients, as many patients in this retrospective cohort did not return for systematic follow-up evaluations; this precluded survival analysis and limits our conclusions to pathological associations rather than prognostic significance. Sixth, the IHC scoring approach focused on maximum-intensity areas, which, while standard practice, may overestimate mean VEGF expression levels across the entire tumor. A direct comparison between maximal-intensity and whole-slide average scoring was not performed in this cohort; given the marked intratumoral heterogeneity in staining that we observed (Table 2), whole-slide averaging would be expected to yield lower IRS values and a correspondingly lower proportion of cases classified as high expression than the 64% reported here—a possibility that should be examined directly in future studies using digital whole-slide quantification. Seventh, the predominance of advanced-stage tumors (72%) may have amplified the associations observed and limits applicability to early-stage populations. Future prospective, multicenter studies with HPV stratification, standardized IHC protocols, and complete survival data are needed to validate and extend these findings.

## 5. Conclusions

The present study demonstrates that high VEGF expression, as assessed by semi-quantitative immunohistochemistry, is significantly associated with advanced tumor stage, higher T stage, and regional lymph node involvement in HNSCC. Multivariate analysis identified nodal status as an independent predictor of high VEGF expression (OR = 2.85; *p* = 0.028), consistent with a possible contributory role of VEGF in nodal spread, although the cross-sectional design of this study precludes causal inference. These findings are consistent with VEGF functioning as a marker of tumor aggressiveness in HNSCC and may support its further evaluation as a candidate target for antiangiogenic strategies in advanced disease, pending prospective validation. Importantly, in the absence of systematic survival data—a consequence of the retrospective design and incomplete long-term follow-up—VEGF cannot be designated a prognostic biomarker based on the present data; its value here is as an indicator of adverse pathological features. Prospective multicenter studies incorporating HPV stratification, standardized IHC protocols, and long-term survival endpoints are required to definitively establish whether VEGF expression holds independent prognostic and predictive value in HNSCC.

## Figures and Tables

**Table 1:** Clinicopathological Characteristics of the Study Cohort.

Variable	Category	*n* (%)
Total patients		50 (100)
Age (years)	Mean ± SD	61.4 ± 9.8
	Range	42–78
Sex	Male	36 (72)
	Female	14 (28)
Tumor location	Larynx	18 (36)
	Oral cavity	14 (28)
	Oropharynx	10 (20)
	Hypopharynx	5 (10)
	Other	3 (6)
Tumor grade	G1	9 (18)
	G2	26 (52)
	G3	15 (30)
Tumor stage (AJCC 8th)	Stage I–II	14 (28)
	Stage III–IV	36 (72)
T stage	T1–T2	16 (32)
	T3–T4	34 (68)
Nodal status	N0	20 (40)
	N+	30 (60)

**Table 2:** Distribution of VEGF Immunoreactivity Scores (IRS).

IRS	*n* (%)
0–1	5 (10)
2–3	13 (26)
4–6	17 (34)
7–9	10 (20)
10–12	1 (10)

**Table 3:** Association between VEGF Expression and Clinicopathological Parameters.

Variable	Category	Low VEGF (*n* = 18)	High VEGF (*n* = 32)	*p*-Value
Age	≤60 years	8 (44.4)	13 (40.6)	0.78
	>60 years	10 (55.6)	19 (59.4)	
Sex	Male	13 (72.2)	23 (71.9)	0.98
	Female	5 (27.8)	9 (28.1)	
Tumor stage	I–II	8 (57.1)	6 (42.9)	0.032
	III–IV	10 (27.8)	26 (72.2)	
T stage	T1–T2	9 (56.3)	7 (43.7)	0.041
	T3–T4	9 (26.5)	25 (73.5)	
Nodal status	N0	11 (55.0)	9 (45.0)	0.018
	N+	7 (23.3)	23 (76.7)	
Tumor grade	G1–G2	13 (44.8)	16 (55.2)	0.089
	G3	5 (33.3)	10 (66.7)	

**Table 4:** Multivariate Logistic Regression Analysis of Predictors of High VEGF Expression.

Variable	OR	95% CI	*p*-Value	VIF
Tumor stage (III–IV vs. I–II)	2.10	0.96–4.62	0.061	2.1
Nodal status (N+ vs. N0)	2.85	1.12–7.24	0.028	2.3
Tumor grade (G3 vs. G1–G2)	1.65	0.72–3.78	0.235	1.3

Nagelkerke R^2^ = 0.24; Hosmer–Lemeshow goodness-of-fit: *p* = 0.41.

**Table 5:** Comparative Summary of Key Studies on VEGF Expression in HNSCC.

Study	*n*	Method	Stage Assoc.	N+ Assoc.	Survival Assoc.
Mineta 2000 [4]	76	IHC	Yes	Yes (*p* = 0.0009)	Yes
Kyzas 2005 [16]	1002 (meta)	IHC (meta)	NR	No (*p* = 0.087)	Yes (HR 1.88)
Tse 2007 [5]	186	IHC	Yes	NR	Yes (multivariate)
Seibold 2013 [26]	98	IHC	Yes	NR	Yes
Zang 2013 [18]	multiple (meta)	IHC (meta)	NR	NR	Yes (HR 1.89)
Schlüter 2018 [19]	n/a	IHC	Stage-dep.	NR	Early only
Siemert 2021 [25]	n/a	Plasma	NR	Yes	Yes
Present study	50	IHC (IRS)	Yes (*p* = 0.032)	Yes (*p* = 0.018)	Not assessed

IHC = immunohistochemistry; NR = not reported; meta = meta-analysis; Stage-dep. = stage-dependent.

## Data Availability

The raw data supporting the conclusions of this article will be made available by the authors on request.

## Abbreviations

The following abbreviations are used in this manuscript:

Abbreviation	Full Term
HNSCC	Head and Neck Squamous Cell Carcinoma
VEGF	Vascular Endothelial Growth Factor
VEGF-A	Vascular Endothelial Growth Factor A
VEGFR-2	Vascular Endothelial Growth Factor Receptor 2
TNM	Tumor–Node–Metastasis
AJCC	American Joint Committee on Cancer
IHC	Immunohistochemistry
FFPE	Formalin-Fixed, Paraffin-Embedded
IRS	Immunoreactivity Score
IS	Intensity Score
PS	Percentage Score
H&E	Hematoxylin and Eosin
HIF-1α	Hypoxia-Inducible Factor 1 Alpha
CI	Confidence Interval
OR	Odds Ratio
SD	Standard Deviation
OS	Overall Survival
DFS	Disease-Free Survival
HPV	Human Papillomavirus
p16	p16 Protein (Surrogate Marker of HPV Status)
VIF	Variance Inflation Factor
EPV	Events Per Variable
IQR	Interquartile Range
SPSS	Statistical Package for the Social Sciences
HR	Hazard Ratio
RR	Relative Risk
DAB	3,3-Diaminobenzidine
κ (kappa)	Cohen’s Kappa Coefficient (Interobserver Agreement)
